# A Case of Poisoning by Oil of Tansy

**Published:** 1867-11

**Authors:** A. C. Blodget

**Affiliations:** Youngville, Pa.


ART. IV—*A Case of Poisoning by Oil of Tansy.* BY A. C. BLODGET, M. D., *Youngville, Pa.*

Case—Mrs. O——, aged about 30, of medium size, nervous temperament, and three months pregnant, took at 5 P. M. August 2d, 1866, fld. ℨiij of oil tanzy. Saw her about three-quarters of an hour afterwards. She was then suffering from nausea and vomiting, which, having been promoted by copious draughts of warm water, had continued freely for the last twenty minutes. The pulse was 110, small and feeble, surface cool and moist, intellect confused with tendency to stupor, breathing much distressed, and clonic spasms appeared. There was moaning and a general appearance of distress, but she acknowledged no pain. The stomach having been apparently well emptied, she was ordered an ounce of castor oil with an equal amount of whisky, to be followed with whisky and milk, equal parts, as freely as the stomach would bear, until the pulse rose. Also sinapisms over the stomach and feet, and friction with hot water and capsicum along the spine and limbs, as freely as practicable. Within an hour and a quarter from taking the drug she became completely unconscious, with pulse almost extinct; skin cold and clammy, breathing very laborious, and the spasms violent at intervals, varying from five to ten minutes. This condition continued between three and four hours, when copious diuresis occurred, followed, in a few minutes, by free alvine discharges. Within the next half hour the oppressive influence of the poison seemed wearing off. The pulse rose a little, the breathing became less labored, and the surface warmer. Directed the use of the whisky to be lessened, and discontinued as soon as reaction was decided. To use mucilaginous drinks, and morphine as far as necessary to control subsequent pain and restlessness. At 9 o'clock next morning there appeared a general improvement; pulse 90, rather small and quick, intellect clear, free from spasm, some pain in the stomach and bowels with tenderness, occasional nausea and slight headache. Treatment continued; i.e., morphine sufficient to keep pain and restlessness well subdued, and mucilaginous drinks. From this time the case progressed favorably and was convalescent in about three days from the beginning. The quantity of whisky taken during the stage of depression, was about six ounces, and probably one-fourth of this was vomited. The condition of the patient's stomach together with the difficulty of inducing her to swallow, rendered a more free use of the stimulus impracticable, if it had been desirable. Subsequently, when questioned, the woman insisted that she felt no pain after taking the tansy, until she emerged from the stupor; that about ten minutes after taking it she felt faint and sick at the stomach, and a feeling of numbness with a sensation as though her arms and legs were suddenly swelling. About this time vomiting commenced, her intellect became confused and she remembered nothing more distinctly until next morning. The drug produced no perceptible effect on the uterus, and the woman was subsequently delivered at full time of a healthy child.

